# Allelic imbalance metre (Allim), a new tool for measuring allele-specific gene expression with RNA-seq data

**DOI:** 10.1111/1755-0998.12110

**Published:** 2013-04-25

**Authors:** Ram Vinay Pandey, Susanne U Franssen, Andreas Futschik, Christian Schlötterer

**Affiliations:** * Institut für Populationsgenetik, Vetmeduni ViennaVeterinärplatz 1, A-1210, Vienna, Austria; † Institut für Evolution und Biodiversität, Universität MünsterD-48149, Münster, Germany; ‡ Institut für Statistik, University of ViennaUniversitätsstr. 5/9, A-1010, Vienna, Austria

**Keywords:** allele-specific gene expression, allelic imbalance, gene expression, mapping bias, RNA-seq

## Abstract

Estimating differences in gene expression among alleles is of high interest for many areas in biology and medicine. Here, we present a user-friendly software tool, Allim, to estimate allele-specific gene expression. Because mapping bias is a major problem for reliable estimates of allele-specific gene expression using RNA-seq, Allim combines two different strategies to account for the mapping biases. In order to reduce the mapping bias, Allim first generates a polymorphism-aware reference genome that accounts for the sequence variation between the alleles. Then, a sequence-specific simulation tool estimates the residual mapping bias. Statistical tests for allelic imbalance are provided that can be used with the bias corrected RNA-seq data.

## Introduction

After microarrays revolutionized, the analysis of gene expression (Schena *et al*. 1995), 2nd-generation-sequencing-based transcriptome profiling has become the method of choice (Garber *et al*. 2011; Ozsolak & Milos 2011). This RNA-seq technique does not only offer the advantage of a higher sensitivity than microarrays, but also provides information about the expression levels of different isoforms (Trapnell *et al*. 2010). Because RNA-seq provides the sequence of individual reads, it is possible to distinguish alleles and thus to estimate allele-specific gene expression (ASE). Given the importance of ASE for understanding variation in cis-regulatory effects, there is considerable interest in tools that provide reliable estimates of allelic imbalance in gene expression (Rozowsky *et al*. 2011; Skelly *et al*. 2011; Turro *et al*. 2011; Satya *et al*. 2012).

Probably the biggest challenge for accurate estimates of ASE comes from the fact that reads from both alleles are mapped against a common reference. If one of the alleles is more similar to the reference than the other one, this results in an unequal success rate of read mapping (mapping bias) (Degner *et al*. 2009; Kofler *et al*. 2011).

Several studies exist that propose frameworks to identify allele-specific gene expression from RNA-seq data (Rozowsky *et al*. 2011; Skelly *et al*. 2011; Turro *et al*. 2011; Graze *et al*. 2012; Satya *et al*. 2012; Shen *et al*. 2012). However, only two studies, AlleleSeq (Rozowsky *et al*. 2011) and MMSEQ (Turro *et al*. 2011) provide a software pipeline, which can be used by researchers to conduct similar analysis. In accordance with the proposed frameworks for ASE identification, both software tools generate a polymorphism-aware diploid genome as a reference for read mapping to reduce the mapping bias. However, their usage is limited to specific data sets. AlleleSeq does not infer polymorphisms between parental genomes, but requires these polymorphisms as input (Rozowsky *et al*. 2011). MMSEQ allows polymorphism detection on the RNA-seq data directly, but requires phasing of genotype calls prior to reconstruction of the parental haplotypes (Turro *et al*. 2011). Both software tools use Bowtie (Langmead *et al*. 2009b) for short read mapping, which does not support gapped alignments, split mapping and SNP aware mapping. Furthermore, the statistical framework of AlleleSeq does not account for replicate data, and neither tool considers a residual mapping bias.

Here, we introduce a new comprehensive and user-friendly software tool, Allim, for measuring allele-specific gene expression in F1 individuals, which accounts for the inevitable mapping bias by combining two strategies. First, a polymorphism-aware diploid reference genome is constructed from parental RNA or genomic short read data. Second, a sequence-specific simulation tool estimates the residual mapping bias. Furthermore, within Allim, a statistical framework is provided, which includes a correction of the residual mapping bias and can take advantage of replicate data. For optimal short read mapping, Allim uses GSNAP, which is capable of SNP tolerant mapping, split mapping and allows gapped alignments (Wu & Nacu 2010).

## Methods

### Implementation and basic usage

**Fig. 1:**
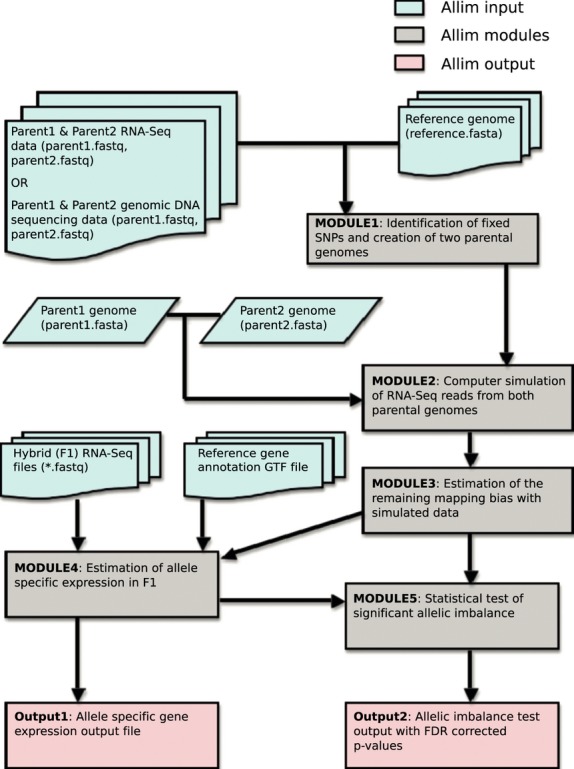
Flowchart of the Allim pipeline. The five modules of the Allim pipeline are (1) Identification of fixed SNPs and creating two parental genomes, (2) Computer simulation of RNA-seq reads from both parental genotypes, (3) Estimation of the remaining mapping bias with simulated data, (4) Estimation of allele-specific expression in F1 and (5) Statistical test of significant allelic imbalance. All these five modules can be run with a single command. All input parameters can be specified in a single configuration file. This configuration file is one of two options. ‘*AllimOptions_2Pexpr* ‘is used when parental genomes have to be generated from parental expression or parental genomic short read data. ‘AllimOptions_2Pgenomes’ is used when two parental genomes are available.

The Allim pipeline was developed in Python 2.7.3 (http://www.python.org), R 2.15.0 (http://cran.r-project.org/) and other third-party packages/tools (Allim user manual; Appendix S1). The Allim pipeline consists of five modules (Fig. 1), which can be run with one single command. All parameter settings can be specified in a single configuration file. An extensive user manual covering all important aspects including dependency installation, Allim usage, sample input, validation, benchmarking and detailed step-by-step description of Allim pipeline is given in Appendix S1 (Allim user manual). The Allim pipeline runs on Mac OS, Linux and other Unix-like operating systems.

### Allim input requirements

Allim determines ASE in F1 individuals and requires SNP information from both parents and RNA-seq data from the F1 individual. However, in order to be applicable to a broader range of experimental designs, Allim offers several options to provide parental information.

1. RNA-seq data from both parents/parental lines
2. DNA-seq data from both parents/parental lines
3. Two parental genomes in FASTA format.

Options 1 and 2 further require a reference genome as input. Fixed SNPs between both parents are determined in Module 1 (Fig. 1) and two parental genomes are created. If no reference genome is available, option 1 can also be used with a reference transcriptome. If option 3 is chosen, Module 1 of the pipeline is skipped.

### Improving the reference genome

**Fig. 2:**
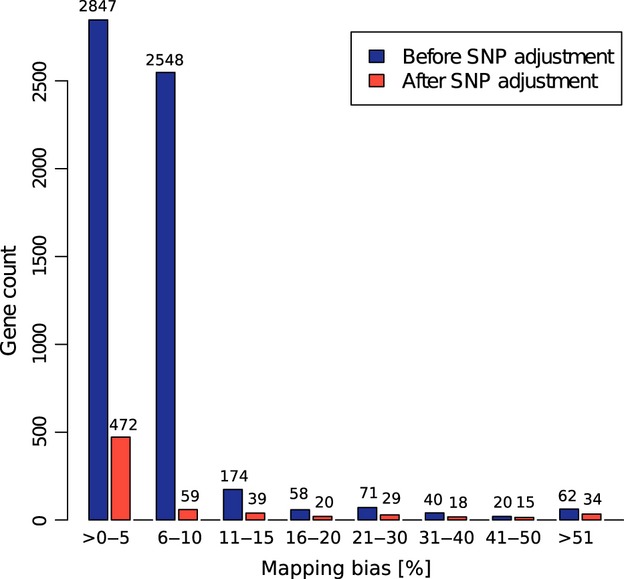
Distribution of gene counts with percent mapping bias. In *Drosophila pseudoobscura,* approximately 96% of all genes (5820) show a residual mapping bias before SNP adjustment (blue bar), whereas only 11% of all genes (686) show a residual mapping bias after SNP adjustment (red bar). Biased genes show mapping biases of various strengths. In both cases, the majority of the biased genes 96% before and ∼69% (472 genes) after the SNP adjustment show only a weak residual mapping bias of ≤5%. The reduction in genes with mapping bias before and after SNP adjustment is significant (Fisher's exact test; *P*-value = 1e-06).

We specifically designed Allim to account for the well-described mapping bias. Recently, it has been shown that the inclusion of polymorphism data in the reference significantly improves the mapping of reads to the reference genome (Satya *et al*. 2012). Most importantly, this strategy is superior to masking polymorphic sites (Degner *et al*. 2009). As a first step, Allim uses GSNAP (Wu & Nacu 2010) to map either genomic DNA or RNA-seq reads from both parents to an available reference genome. Based on the mapped reads, fixed SNPs between both parents are identified. To increase mapping success of reads, the fixed SNPs are used to create a polymorphism-aware genome via GSNAP, which is used as a reference genome in a subsequent round of read mapping. This procedure of read mapping, fixed SNP calling and construction of an improved polymorphism-aware genome via GSNAP can be iterated to fine-tune the identification of fixed SNPs. In case two parental genomes are available, these can be used directly. Consistent with previous results (Satya *et al*. 2012), we find that the modified reference genome improves the mapping success and reduces the extent of mapping bias (Table 1, Fig. 2).

**Table 1:** Improvement of mapping success via genome modification (SNP inclusion). The performance of Allim was validated with experimental as well as simulated RNA-seq reads. The experimental data consisted of paired-end RNA-seq reads from males and females of two different isofemale lines (ps88 and ps94) of *Drosophila pseudoobscura* (Table 2). (An ‘isofemale line’ is established by a single female, typically caught and inseminated in the wild. Due to inbreeding over multiple generations, genetic heterozygosity in the line is reduced.) For the experiment, male and female flies from both lines were pooled and sequenced. Via Module 1 fixed SNPs between both parental lines were identified and used to create two parent-specific genomes. The simulation of reads was based on the two parental genomes (see Methods). The two parental genomes are later used as a reference to map F1 offspring RNA-seq reads

			Mapped single reads (%)				Improvement (% of total number of reads)	
					
	Total number of single reads		Before SNP adjustment		After SNP adjustment			
		
RNA-Seq data	No. of reads p88	No. of reads p94	ps88	ps94	ps88	ps94	ps88	ps94
Female data	79 981 000	79 998 000	91.18	92.15	91.49	92.22	0.31	0.07
Male data	76 877 000	79 207 000	85.97	86.73	86.19	87.05	0.22	0.32
Simulated data	122 682 000	122 682 000	90.94	90.96	91.05	91.03	0.11	0.07

### Quantification of ASE and assessment of allelic imbalance

Previous benchmark tests of split read mappers consistently found that GSNAP is one of the most reliable mapping tools for RNA-seq data (Grant *et al*. 2011). Furthermore, GSNAP is designed to account for polymorphisms when mapping reads against a reference (Wu & Nacu 2010). Allim quantifies ASE for F1 individuals by determining the number of reads that can be unambiguously assigned to one of the parental genotypes. The unit, for which expression strength is measured, can be either an entire gene or a single exon (paired-end reads mapping to the same focal region are only counted once even if they span multiple SNPs). Thus the later option allows testing for allelic differences in isoform representation.

### Correcting the residual mapping bias by computer simulations

While the reconstruction of the two parental genotypes substantially reduced the mapping bias, we use computer simulations to estimate the residual mapping bias. A grid of RNA-seq reads from both parental alleles are generated using the two genomes (i.e.: 2 × 100 bp paired ends with 78 bp insert size). For each polymorphic site, the same number of reads is generated for both genomes. Thus, in absence of a mapping bias, all genes should have an expression ratio of one. In contrast to this expectation, we observe for *Drosophila pseudoobscura,* a residual mapping bias for about 11% of the genes. Most of those biased genes show a weak bias ≤5%, while strong residual biases are limited to relatively few genes (Fig. 2). Reasoning that the experimental RNA-seq reads experience the same mapping bias, we propose to correct for this residual mapping bias before we test for statistical significance of allelic imbalance. We further compared the mapping bias before SNP adjustment and after SNP adjustment with the same simulated data set. We show that the mapping bias has been reduced significantly with SNP adjustment (Fig. 2).

### Statistical tests for allelic imbalance

To assess the statistical significance of allelic imbalance for samples without biological replication for each gene (exon), Allim relies on the *G*-test. This test may, however, overstate the statistical significance, as some sources of variation can only be taken into account when replicates are available. Allim, therefore, also provides analysis of variance (ANOVA) tests for allelic imbalance across replicates. Both approaches inherently account for different library sizes and are complemented with two additional scaling factors. The residual mapping bias is integrated via the observed expression ratios of the simulated data. Additionally, libraries are normalized via the TMM factor (Robinson & Oshlack 2010). The TMM normalization eliminates biases in the data due to technical differences between the samples or vast expression changes of a few genes under only one condition that can affect expression ratios for all remaining genes.

### Allim validation

We used RNA-seq data from two different *D. pseudoobscura* isofemale lines (library sizes: ∼80 million read pairs, 2 × 100 bp, insert size 78; Table 2) and ran our Allim pipeline after pooling the reads from both libraries. In contrast to experiments measuring allelic imbalance, the origin of each read is known, which allowed us to measure the performance of Allim.

**Table 2:** Number of paired-end RNA-seq reads of *Drosophila pseudoobscura* used for Allim validation. The data was generated on an Illumina GA IIx sequencer. The *Drosophila pseudoobscura* isofemale lines ps94 (stock number 14011-0121.94) and ps88 (stock number 14011-0121.88) were obtained from the UC San Diego Drosophila Stock Center. Flies were reared on standard cornmeal-molasses-yeast-agar medium and maintained at 19 °C under constant dark conditions. For each line, virgin females and virgin males were collected from 15 to 20 replicate vials, pooled and allowed to age for 3–7 days before shock-freezing in liquid nitrogen (Palmieri *et al*. 2012)

Samples	Read pairs (in millions)	Insert size (bp)	Read length (bp)
ps88 males	79.21	78	100
ps88 females	80.00	78	100
ps94 males	79.21	128	100
ps94 females	80.00	68	100

Our results show that accounting for the sequence divergence of the two lines allowed to map on average 0.23% more reads (Table 1) and reduced the total mapping bias from 69 to 11% of all genes (Fig. 2). Furthermore, Allim assigns between 98.60 and 99.97% of the reads containing fixed SNPs to the correct parental allele (Table 3). Simulated reads were assigned slightly better (99.99%) (Table 3). We attribute this difference to confounding signals of SNPs, which are not fixed between the two isofemale lines.

**Table 3:** RNA-Seq data sets used to test accuracy of Allim to identify the parental origin of a read. The experimental data consisted of paired-end RNA-seq reads from males and females of two different isofemale lines of *Drosophila pseudoobscura* (Table 2). It can be seen that the experimental reads from line p88 were more often correctly identified by the pipeline. The slight discrepancy between the two strains reflects the fact that ps94 is derived from the strain that was used to generate the *D. pseudoobscura* reference genome

Data set	No. of correctly identified reads, ps88 (%)	No. of correctly identified reads, ps94 (%)
Pooled reads from females of both lines	99.97	98.96
Pooled reads from males of both lines	99.96	98.60
Simulated reads for both parental genomes	99.99	99.99

### Comparison with similar tools

Two similar tools to assess allele-specific expression are freely available: AlleleSeq (Rozowsky *et al*. 2011) and MMSEQ (Turro *et al*. 2011). In comparison with these tools, Allim includes several advanced and user-friendly features.

### Input requirements and inference of parental variants

AlleleSeq requires polymorphism data in form of family trios as input, which consist of SNP information of the reference allele, the maternal, paternal and child genotypes along with phase information. MMSEQ calls genotypes for every given individual from the sequence data itself. The phase can then be estimated with the integrated software tool polyHap (Su *et al*. 2010). As the phase information has to be imputed from the genotype data, complete and accurate phasing results can only be obtained when input data for a large number of individuals are provided. Allim also determines SNP information from the provided sequence data directly, which can either be transcriptome or genome data from both parents. Alternatively, genome sequences of both parents can be provided. In both cases, the full phase of both parental genotypes is known.

### Output options and statistical testing

The final output of MMSEQ is a table of allele-specific expression counts on either gene or isoform level. To assess allelic imbalance, this table can be used with any statistical test or software tool based on raw count data. Allim similarly produces expression tables for allele-specific expression on the gene and the exon level. Additionally, Allim has implemented two statistical tests (*G*-test, ANOVA) to assess allelic imbalance, while accounting for the residual mapping bias. The tests for allelic imbalance provide p-values and FDR corrected q-values per gene or exon. In contrast to the gene/exon-wise approach, AlleleSeq assesses allelic imbalance for every heterozygous SNP. Statistical significance is assessed via a binomial *P*-value assuming 50/50 probability including FDR correction. Important features of Allim and the other two available tools are given in Table 4.

**Table 4:** Comparison of various features of Allim to other available tools

Features	AlleleSeq*	MMSEQ†	Allim
Inference of parental variants	No	Yes	Yes
Construction of polymorphism-aware diploid genome	Yes	Yes	Yes
Estimation and integration of residual mapping bias	No	No	Yes
Statistical test for Allelic imbalance	Yes	No	Yes
Use replicate information for statistical testing	No	Not applicable	Yes
ASE exon wise/per isoform	No	Yes	Yes
Mapper used	Bowtie	Bowtie	GSNAP
Single command to run whole pipeline	No	No	Yes

* Rozowsky *et al*. (2011).

† Turro *et al*. (2011).

## Conclusion

Allim is an open-source and user-friendly tool, which estimates allele-specific gene expression in F1 crosses. It provides an integrated pipeline for estimating the allele-specific gene expression and allelic imbalance tests. Compared to other available software tools, Allim provides a range of additional features and allows for a wide range of input options.

## Obtaining Allim

Allim requires Python 2.7.3, R 2.15.0 and other third-party tools and works on all Unix operating system. The source code, user manual and test data sets are available online from http://code.google.com/p/allim/.

## Supporting Information

**Appendix S1.** Allim User Guide, version 1.0.
