# Supplementary material for: Allelic imbalance metre (Allim), a new tool for measuring allele-specific gene expression with RNA-seq data
Source: Mol Ecol Resour. 2013 Apr 25;13(4):740–5. doi: 10.1111/1755-0998.12110 (PMC3739924; doi:10.1111/1755-0998.12110)
Supplement: Supplementary file 1 [file men0013-0740-SD1.pdf]

Allim User Guide, version 1.0

Institut für Populationsgenetik
Vetmeduni Vienna
Veterinärplatz 1, A-1210 Wien
Austria
March 18 2013

Contents

1. Introduction

2. System Requirements

3. Allim Installation

4. The Validation of the Installation & Sample Input Files

5. Allim Input Description

6. Allim Modules Description

7. Allim Output / Results Files

8. Benchmark of the Allim Pipeline

9. Validation

10 Contact Information

11. References

1. Introduction

Allim, Allelic imbalance meter, offers an integrated and user-friendly solution for measuring allele specific gene expression (ASE) within species. Allim estimates allelic imbalance in F1 hybrids. Since mapping bias is the largest problem for reliable estimates of allele specific gene expression using RNA-seq, Allim combines two different measures to account for mapping biases. First, Allim generates a polymorphism aware reference genome that accounts for the sequence variation between the alleles of both parents (or parental lines). Second, Allim includes a sequence specific simulation tool to estimate the remaining mapping bias. This estimated mapping bias is then incorporated in the statistical tests for allelic imbalance.

The pipeline requires whole transcript high throughput RNA sequencing (RNA-seq) reads of F1 hybrids. Additionally, either RNA-seq reads for both parents, genomic sequencing reads for both parents or two parental genomes for both parents have to be provided in separate files. Allim was tested on Illumina paired-end RNA-seq reads but it can also handle FASTQ files from other NGS sequencing platforms. The provided parental RNA-seq libraries can be from homozygous as well as heterozygous parents (however, all heterozygous loci will be excluded during the analysis).

Allim has five modules that can be run by a single command. All input parameters can be specified in the AllimOptions file. These parameters are then used to run the complete pipeline. Allim provides two different input options:

AllimOptions_2Pexpr: This configuration file can be used when RNA-seq data for parent1, parent2 and F1 (hybrid) is provided. In this case Allim uses parent1 and parent2 RNA-seq reads to identify the fixed SNPs. The fixed SNPs are subsequently used to generate two "parental -genomes", one for each parent. Note, rather than RNA-seq data, also DNA reads can be provided for the two parents.

With Allim if user does not have reference genome and corresponding gene annotation in GTF format. It is possible to provide a single reference transcriptome along with RNA-seq data of both parents. The use of a reference transcriptome (or contigs from a RNA-Seq de novo assembly) instead of a reference genome is accompanied by the following differences:

- In a transcriptome assembly different isoforms are typically presented by different contigs. Furthermore, assemblies often contain additional redundancies between contigs due to sequencing errors and polymorphisms in the RNA-seq reads used for de novo transcriptome assembly.
- As Allim creates two parental references (genomes or here transcriptomes), and maps

RNA-seq reads of the F1 individual to the "diploid genome", only reads that map non-ambiguously can be used to determine ASE profiles. It is therefore recommended to remove redundancy from the transcriptome assembly before Allim usage.

- As transcriptome assemblies typically do not have a gtf file containing gene features (needed as Allim input). We have therefore added an additional short script to obtain a simple gtf file based on contig ids.

AllimOptions_2Pgenomes: This configuration file can be used when the user provides RNA-seq data for the F1 hybrid and a genomic sequence for each parental line. If genomic sequences of both parents are provided, both FASTA files need to have the same size and identical FASTA IDs.

Five Modules of Allim:

(1) Identification of fixed SNPs
(2) Computer simulation of RNA-seq reads with fixed SNPs
(3) Estimation of the remaining mapping bias with simulated data
(4) Estimation of allele specific expression for experimental data
(5) Statistical test of significant allelic imbalance

The source code and the user manual of Allim are available at http://code.google.com/p/allim/

2. System Requirements

To use the Allim pipeline, the user needs to meet the following requirements:

1. Linux or Macintosh OSX system or any other Unix 64 bit system with at least 4 GB of RAM and 2 CPU (processors)
2. Python 2.7.3 [http://www.python.org/download/]
3. Biopython 1.59 or higher [http://biopython.org/wiki/Download]
4. NumPy [http://numpy.scipy.org/]
5. PICARD (BuildBamIndex.jar, MergeSamFiles.jar, SortSam.jar) [http://sourceforge.net/projects/picard/files/picard-tools/]
6. SAMTOOLS [http://sourceforge.net/projects/samtools/files/samtools/]
7. BedTools (bamToBed, intersectBed, sortBed) [http://code.google.com/p/bedtools/]
8. GSNAP [http://research-pub.gene.com/gmap/]
9. R version 2.15.0 [http://cran.r-project.org/]

10. RPy 2.2.2 [http://rpy.sourceforge.net/rpy2.html]
11. R package ***car*** version 2.0-12 [http://cran.r-project.org/web/packages/car/index.html]
12. R package ***multcomp*** version 1.2-12 [http://cran.r-project.org/web/packages/multcomp/index.html]
13. Bioconductor package ***edgeR*** [http://www.bioconductor.org/packages/2.10/bioc/html/edgeR.html]
14. Bioconductor package ***limma*** [http://www.bioconductor.org/packages/release/bioc/html/limma.html]

Note: To install R packages listed above 11-14, R >=2.15.0 should be installed.

2.1 Operating system:

The Allim package is designed to work with a 64-bit Unix operating system with at least 4 GB of RAM and 2 CPUs (processors).

2.2 Python installation:

Allim is developed on Python version 2.7.3. Python 2.7.3 for your operating system can be obtained from http://www.python.org/download/. After the download follow the instructions given on the web page to complete the python installation and configuration.

2.3 Biopython installation:

Allim requires biopython for efficient fasta sequence reading, writing and other sequence manipulations. Once python is installed, biopython can be obtained and installed with the following steps:

Step1: Download the biopython source code from http://biopython.org/DIST/biopython-1.60.tar.gz

Step2: Uncompress the downloaded file with the following command:

tar –zxvf biopython-1.60.tar.gz

This command will return the folder/directory *biopython-1.6.0*

Step3: Enter the uncompressed folder with following command:

cd biopython-1.60

Step4: To install the biopython package run these two commands:

python setup.py build

python setup.py install

2.4 NumPy installation:

NumPy is the fundamental package for scientific computing with Python. In the Allim pipeline NumPy is used to integrate sequencing errors during the simulation of RNA-seq reads. In order to install NumPy on any Unix system the following steps are required:

Step1: Download the NumPy package source code from

http://sourceforge.net/projects/numpy/files/NumPy/1.6.0/

Step2: Uncompress the downloaded file with following command:

tar –zxvf numpy-1.6.0.tar.gz

This command will return the folder/directory *numpy-1.6.0*

Step3: Enter the uncompressed folder with following command:

cd numpy-1.6.0

Step4: To install this python package run these two commands:

python setup.py build

python setup.py install

2.5 PICARD installation:

Picard comprises Java-based command-line utilities that manipulate SAM/BAM files. It is a collection of many JAVA jar files, which can be downloaded and used directly without prior installation.

Step1: Download the latest version of PICARD from

http://sourceforge.net/projects/picard/files/picard-tools/1.75/

Step2: Uncompress the downloaded file with the following command:

unzip picard-tools-1.75.zip

The Allim pipeline uses three jar files: 1) *BuildBamIndex.jar*, 2) MergeSamFiles.jar, 3) *SortSam.jar*. Provide the full paths of these three jar files in AllimOptions run file to run the Allim pipeline.

2.6 SAMTOOLS installation:

SAMTOOLS provide various utilities for the manipulation of alignments in the SAM format, including sorting, merging, indexing and generating alignments in a per-position format.

Step1: Download latest SAMTOOLS source code from

http://sourceforge.net/projects/samtools/files/samtools/

Step2: Uncompress the downloaded file samtools-0.1.18.tar.bz2 with following command:

tar -xvjf samtools-0.1.18.tar.bz2

This command will return the folder/directory *samtools-0.1.18*

Step3: Enter the uncompressed folder *samtools-0.1.18* with following command:

cd samtools-0.1.18

Step4: To make samtools executable run the make command:

make

After running the make command an executable called samtools will be created. Provide the full path of the samtools executable in the AllimOptions run file to run the Allim pipeline.

2.7 BedTools installation:

BedTools provide a set of functions to sort and intersect various genomic formats including bam files and files that contain genomic annotation such as gene position and SNP information.

Step1: Download BedTools from

http://bedtools.googlecode.com/files/BEDTools.v2.16.2.tar.gz

Step2: Uncompress the downloaded file BEDTools.v2.16.2.tar.gz with the following command:

tar -xzvf BEDTools.v2.16.2.tar.gz

This command will return the folder/directory *BEDTools-Version-2.16.2*

Step3: Enter the uncompressed folder *BEDTools-Version-2.16.2* with following command:

cd BEDTools-Version-2.16.2

Step4: To make BedTools executable run the make all command:

make all

After running the "make all" command, *BEDTools-Version-2.16.2/bin* sub-folder/sub-directory will be created, which contains all BedTool executables.

The Allim pipeline uses three executables: 1) intersectBed, 2) sortBed, 3) bamToBed. Provide the full paths of these three executables in the AllimOptions run file to run the Allim pipeline.

2.8 GSNAP installation:

GSNAP (Genomic Short-read Nucleotide Alignment Program) is a mapper for RNA-seq data. It has the advantages that it can detect splicing events and is capable of SNP tolerant alignments (Wu & Nacu 2010).

Step1: Download the GSNAP source code from

http://research-pub.gene.com/gmap/src/gmap-gsnap-2012-07-20.tar.gz

Step2: Uncompress the downloaded file "*gmap-gsnap-2012-07-20.tar.gz*" with following command:

tar -xzvf gmap-gsnap-2012-07-20.tar.gz

Step3: Enter the uncompressed folder *"gmap-2012-07-20"* with following command:

cd gmap-2012-07-20

Step4: To install the gmap package run these four commands:

./configure

make

make check (optional)

make install

The above four commands will build GSNAP and other executables in "*/usr/bin*"

Step5: To check the GSNAP installations run this command:

gsnap –help

This command will return the detailed help manual for various GSNAP parameters if the installation was successful.

Note: For more detailed information how to install GSNAP please read the *gmap-2012-07-20/README* file.

2.9 R installation:

The R source code specific to your operating system can be obtained from http://cran.r-project.org/. To install R, please follow the instructions provided with R.

2.10 RPy2 installation:

RPy2 is a python class designed to do statistics programming using R in python. In order to install RPy2 on any Unix system the following steps are required:

Step1: Download the RPy2 package source code from

http://sourceforge.net/projects/rpy/files/rpy2/2.2.x/

Step2: Uncompress the downloaded file with following command:

tar –zxvf rpy2-2.2.2.tar.gz

This command will return the folder/directory *rpy2-2.2.2*

Step3: Enter the uncompressed folder with following command:

cd rpy2-2.2.2

Step4: To install the RPy2 package run the following two commands:

python setup.py build

python setup.py install

2.11 Installation of the R package *car*:

Step1: Download the R package *car* source code from

http://cran.r-project.org/src/contrib/car_2.0-12.tar.gz

Step2: Open the terminal and run the following command:

R CMD INSTALL car_2.0-12.tar.gz

2.12 Installation of the R package *multcomp*:

Step1: Download R package *multcomp* source code from

http://cran.r-project.org/src/contrib/multcomp_1.2-12.tar.gz

Step2: Open the terminal and run the following command:

R CMD INSTALL multcomp_1.2-12.tar.gz

2.13 Installation of the R package *edgeR*:

Step1: Download R package *edgeR* source code from

www.bioconductor.org/packages/2.3/bioc/src/contrib/edgeR_1.0.4.tar.gz

Step2: Open the terminal and run following command:

R CMD INSTALL edgeR_1.0.4.tar.gz

2.14 Installation of the R package *limma*:

Step1: Download R package *limma* source code from

http://phase.hpcc.jp/mirrors/stat/R/CRAN/src/contrib/limma_2.0.2.tar.gz

Step2: Open the terminal and run the following command:

R CMD INSTALL limma_2.0.2.tar.gz

3. Allim Installation

The user can download the latest version of Allim from http://code.google.com/p/allim/. The file to download is called Allim_1.0.tar.gz. Move the file to an appropriate directory and run the following command to uncompress the file:

tar –zxvf Allim_1.0.tar.gz

Note that after uncompressing the tar.gz file, a new folder will be created named *Allim_1.0*. This directory contains the following files:

< Allim_1.0>

|

- <Allim.py>
- <AllimOptions_2Pexpr>
- <AllimOptions_2Pgenomes>
- <classes>

|

- <ase.py>
- <call_gsnap.py>
- <fixed_snp.py>
- <gff2gtf.py>
- <input_parse.py>
- <inter_species.py>
- <simulateRNAseq.py>
- <simulation.py>
- <utility.py>
- < statistical_test.py>

■ <executables>

|

- <bamToBed>
- <intersectBed>
- <sortBed>
- <samtools>
- <BuildBamIndex.jar>
- <MergeSamFiles.jar>
- <SortSam.jar>

4. The Validation of the Installation & Sample Input Files

To validate the installation of the Allim pipeline it can be run with a small test data set. The test data set and the corresponding Allim configuration files can be obtained from the following URLs:

Parent1 RNA-seq: http://allim.googlecode.com/files/parent1_RNAseq_fastq.tar.gz

Parent2 RNA-seq: http://allim.googlecode.com/files/parent2_RNAseq_fastq.tar.gz

F1 RNA-seq: http://allim.googlecode.com/files/F1_RNAseq_fastq.tar.gz

Parent1 genome: http://allim.googlecode.com/files/parent1_genome.fa

Parent2 genome: http://allim.googlecode.com/files/parent2_genome.fa

Reference fasta file: http://allim.googlecode.com/files/reference.fa

Gene annotation file: http://allim.googlecode.com/files/reference.gtf

Allim configuration file: http://allim.googlecode.com/files/AllimOptions_2Pexpr

Allim configuration file: http://allim.googlecode.com/files/AllimOptions_2Pgenomes

Note: You either need to use the parental RNA-seq or the parental genomic data. The option you choose depends on the type of input data you have for your own analysis.

Create a folder named “test_data” in the Allim_1.0 directory and download all the above test data files into this folder, unzip and extract the compressed files. Move the *AllimOptions_2Pexpr* and *AllimOptions_2Pgenomes* files into the directory Allim_1.0 directly. The Allim pipeline can then be run on the test data set with the following two steps:

1. Open the terminal and enter the *Allim_1.0* directory.

   cd Allim_1.0

2. Run the Allim pipeline with the test data set via the following command (the type of the AllimOptions file that is used depends on the type of your input data):

   python Allim.py --option-file AllimOptions_2Pexpr

   OR

   python Allim.py --option-file AllimOptions_2Pgenomes

To get help on how to run Allim and required parameters enter:

python Allim.py –help

5. Allim Input Description

Allim can be run with the following command, which should be run under the *Allim_1.0* directory:

python Allim.py --option-file <path to AllimOptions file>

However, before running Allim with your own dataset all parameters have to be specified in the appropriate Allim Options files (AllimOptions_2Pexpr OR AllimOptions_2Pgenomes).

Global input parameters:

Figure 1 shows global input parameters, which are essential for all modules of Allim. Figure 2 shows how the paths to third party tools that are used by Allim can be specified in the AllimOptions file.

```
# Output directory: the name of the directory can be changed.
# The directory will be generated in <Allim_1.0>
OUTPUT_DIRECTORY = /Allim_1.0/test_output

## Give the number of replicates your data consists of.
REPLICATE_COUNT=1

# Give the path to the fasta file of the genomic reference.
REFERENCEE_FASTA = /Allim_1.0/test_data/reference.fa

# Give the gene annotation file of your genomic reference in GTF format.
REFERENCE_GTF_FILE = /Allim_1.0/test_data/reference.gtf

# Give the number of processors that should be used for read mapping.
THREAD = 15

# Give the minimum base quality required to call a fixed SNP and to use a nucleotide position for
# the assessment of allele specific expression profiles. The base quality ranges between 0-40.
# The sequencer for each nucleotide assigns it in read sequence.
MINIMUM_BASE_QUALITY = 20

#
# GSNAP or any other short read mappers (BWA, Bowtie, TopHat)
# assign a mapping quality to each mapped read. It ranges between 0-40.
# Give the minimum mapping quality of a read required to be included in the analysis.
MINIMUM_MAPPING_QUALITY = 20

#
# Specify the encoding scheme for the base quality of the sequence reads.
# This can either be "sanger" or "illumina". This parameter value is case sensitive.
# Note:
# "sanger" (Illumina 1.3+ Phred+33, raw reads typically (0, 40))
# "illumina" (Illumina 1.5+ Phred+64, raw reads typically (3, 40))
QUALITY_ENCODING= illumina
```

Figure 1: Global input parameters of the Allim pipeline. The figure shows an extract from the AllimOptions_2Pexpr file. In AlimOptions_2Pgenomes, the parameter REFERENCEE_FASTA is replaced by the two parameters: PARENT1_REFERENCEE_FASTA AND PARENT2_REFERENCEE_FASTA.

```
##########################################################################################################
#
# Third party software/tool executables
# Provide the full path to the third party executables to run the Allim pipeline.
#
#
##########################################################################################################


### Get samtools to manipulate SAM and BAM files from http://samtools.sourceforge.net/
# Provide the full path of the executables relative to the <Allim_1.0>
SAMTOOLS = /Allim_1.0/executables/samtools


### Get intersectBed executables from a collection of useful utilities called bedtools-
### from http://code.google.com/p/bedtools/
# Provide the full paths of the executables relative to the <Allim_1.0>
INTERSECTBED = /Allim_1.0/executables/intersectBed
SORTBED = /Allim_1.0/executables/sortBed
BAMTOBED = /Allim_1.0/executables/bamToBed


#### Three PICARD JAR files from http://picard.sourceforge.net/index.shtml
# Provide the full paths of the executables relative to the <Allim_1.0>
SORTSAM = /Allim_1.0/executables/SortSam.jar
MERGESAMFILES = /Allim_1.0/executables/MergeSamFiles.jar
BUILDBAMINDEX = /Allim_1.0/executables/BuildBamIndex.jar
```

Figure 2: Specifications of full paths of external software/tools used in Allim.

The remaining input parameters are described in the following chapter along with the Allim modules description.

6. Allim Modules Description

Module 1: Identification of fixed SNPs

This module is only used when the input option, "AllimOptions_2Pexpr" is chosen (RNA-seq or genomic DNA reads of both parents are provided). With the input option, "AllimOptions_2Pgenomes" (two genomes, one for each parent) is used this module will be skipped.

The module determines fixed SNPs between the parental genotypes (lines) based on the user provided RNA-seq libraries. It accepts multiple replicates for the calculation of fixed SNPs to increase the power and accuracy of SNP detection. For each parental genotype and replicate RNA-seq data (alternatively genomic DNA reads) can be provided for paired-end sequence data (pairs of fastq files, read1.fq and read2.fq, for each condition). Besides the RNA-seq data (genomic reads) further parameters can be specified as shown in Figure 3. The type of data that is used for calling fixed SNPs, either RNA or genomic sequence is reads is specified via the parameter SEQUENCE_TYPE ("mRNA" or "DNA").

In Allim the identification of fixed SNPs is based on the alignments of the RNA-seq reads (alternatively genomic DNA reads) performed with the GSNAP mapper (Wu & Nacu 2010). GSNAP is a unique mapper that can integrate given SNP information into the mapping algorithm in order to improve read mapping of allelic variants. Allim makes use of this functionality by integrating the fixed SNP information into the mapping process to improve the quality of the alignments. However, in the beginning no fixed SNP information is available. Therefore, multiple cycles of read mapping and subsequent calling of fixed SNPs are required to improve the quality of the alignment and the identified fixed SNPs. The user can specify how often this procedure should be repeated (minimum & default: 2 cycles). The number of cycles given as input provides the opportunity to fine-tune SNP calling accuracy, as this is dependent on the accuracy of the alignment. As output a fixed SNP table for each cycle is saved in the given output directory. The fixed SNP information determined in the last cycle is used in subsequent modules.

The parameters MINIMUM_COVERAGE, FIXED_ALLELE_FREQUENCY, MINIMUM_MAPPING_QUALITY and MINIMUM_BASE_QUALITY are used in each cycle to determine fixed SNPs between both parental genotypes (Figure 3).

```
##########################################################################################################
#
# Fixed SNP OPTIONS
# Step1: Identification of fixed SNPs (fixed differences between parental strains)
#        The fixed SNPs will later be used to estimate allele specific gene expression
#        in simulated and experimental data.
#
##########################################################################################################

#### Give the sequencing type. Allim can calculate fixed SNP either with RNA-Seq FASTQ files OR
#### with DNA sequencing FASTQ files from parent1 and parent2. For Fixed SNP identification
#### user can specify the Sequence type: SEQUENCE_TYPE=mRNA or SEQUENCE_TYPE=DNA
#### It is case sensitive to give exact word mRNA or DNA
SEQUENCE_TYPE=mRNA


## Provide the full paths (relative to <Allim_1.0>) of the paired-end sequencing files
## of the parents and all present replicates.
#
## Paired-end sequencing files and insert size (fragment size - 2(read length)) should be
## provided in following format:
# read1.fq,read2.fq,78
# The read1.fq file contains all "read 1" reads of the paired end reads. read2.fq contains
# all corresponding "read 2" reads.
# 78 is the insert. Make sure that the order of the paired end reads is identical in both fastq files.

# The insert size is an optional parameter. Fastq files for both paired-end reads are mandatory.

# For each replicate provide 3 files 1) parent1; 2) parent2; and 3) hybrid (F1)
#### Replicate 1: parent1,parent2 and hybrid fastq file data
PARENT1_FASTQ_FILE = /Allim_1.0/test_data/parent1_read1.fastq,/Allim_1.0/test_data/parent1_read2.fastq,68
PARENT2_FASTQ_FILE = /Allim_1.0/test_data/parent2_read1.fastq,/Allim_1.0/test_data/parent2_read2.fastq,78


#### Replicate 2: parent1,parent2 and hybrid fastq file data
#PARENT1_FASTQ_FILE =
#PARENT2_FASTQ_FILE =

#### Replicate 2: parent1,parent2 and hybrid fastq file data
#PARENT1_FASTQ_FILE =
#PARENT2_FASTQ_FILE =


# Provide parameters for the mapping with GSNAP and the identification of fixed SNPs.
#
# Fixed SNPsbetween both parents are called based on the mapping of the reads from both parents.
# However, the mapping with GSNAP can be improved by integrating the fixed SNP information
# to remap the sequence reads of the parents.

# The following parameter allows to specify the number of iterations that is done to call the final fixed SNPs
# for the subsequent analysis. (The minimum number is 2.)
CALCULATE_FIXED_SNP_ITERATION = 2


# Give the minimum coverage required to call a fixed SNP.
# This means that at least X mapped reads from each parent have to map to the respective nucleotide
# position in the reference (X = minimum coverage).
MINIMUM_COVERAGE = 2

# Give the minimum frequency of a nucleotide at a genomic position in one parent that is-
# required to call a fixed SNP. This frequency to identify the major allele in each parent-
# has to be between 0.51-1.0.
# Note that the choice of this frequency has an effect on the number of reads that can be-
# used to determine allele specific expression (ASE) (power) AND the accuracy to determine ASE.
# higher frequency: lower throughput, higher accuracy
# lower frequency: higher throughput, lower accuracy
FIXED_ALLELE_FREQURNCY = 1.0
```

Figure 3: Input options for the identification of fixed SNPs between the two parental genotypes. The figure shows an extract from the AllimOptions_2Pexpr file.

Module 2: Computer simulation of RNA-seq reads

This module simulates paired-end Illumina reads in fastq format. It internally uses the fixed SNP information to create two "parental genomes", one for each parent or alternatively the user provided reference genomes. Further it used the information from the provided GTF file (provided as a global input parameter).

For the read simulation, first two parent specific genomes ("parental genomes"), which only differ with respect to the identified fixed SNPs, are generated. For each parental genome all possible paired-end reads that cover at least one fixed SNP position are simulated once. Therefore, the expression ratio been reads simulated for each parent should equal **1** for each gene. In contrast, the deviation from an expression ratio of **1** in the simulated data for a gene indicates a remaining mapping bias, which is caused by the mapper itself.

Algorithm:

1. Creation of two parent specific ("parental") genomes via the substitution of the base at a fixed SNP position in initial reference genome (this step is skipped if two parental genomes are provided initially).
2. Construction of the longest transcript for each gene for both parental genomes separately. Due to step 1, the transcripts for both parental genomes only differ at the fixed SNP positions (AllimOptions_2Pexpr). If two parental genomes are provided initially the both genomes must have identical genome length and identical gene annotation. Each transcript consists of the 5'UTR, the CDS and the 3'UTR.
3. Simulation of the reads for both "transcriptomes" (as defined in step 2) separately. This results in the same number of reads from the identical genomic locations for both parents. The simulation is implemented via a sliding window approach where the "simulation window" slides one base pair at a time from 5' towards 3' of transcript. Only the reads that span a fixed SNP are kept as the remaining ones are not informative for the subsequent analysis (they cannot be used to determine ASE). This approach results in equal coverage of SNP position in the middle of a transcript and decreasing coverage of SNPs towards both ends of the transcript (end of a transcript: 2 x read length + insert size).

Additional parameters that define the properties of the simulated reads are shown in Figure 4.

```
##########################################################################################################
#
# Simulation OPTIONS
# Step2: Simulating RNA-Seq paired-end reads for the whole transcriptome.
#        This simulation data will be used to estimate the residual mapping bias after -
#        the generation of a polymorphism aware reference genome.
#
##########################################################################################################

# Give the ASCII letter, which will be assigned to each nucleotide of simulated read in FASTQ file.
# Example: ASCII_QUALITY_LETTER=e for illumina encoding; or ASCII_QUALITY_LETTER=H for sanger encoding.
# For illumina encoding the ASCII QUALITY letters UVWXYZ[\]^_`abcdefgh encode for base qualities from 20-40.
# For sanger encoding the ASCII QUALITY letters 6789:;<=>?@ABCDEFGHI encode for base qualities from 20-40.
# Give a single ASCII letter, which will be assigned for each nucleotide in the simulated read.
ASCII_QUALITY_LETTER = e

# Provide the read length of the simulated reads. For example: READ_LENGTH=100 to simulate 100 bp reads.
READ_LENGTH = 100


# Provide the insert size between read1 and read2 for the paired-end read simulation.
# (fragment size = insert size + 2 * read length)
INSERT_SIZE = 78
```

Figure 4: Input options for the computer simulation of RNA-seq reads from two parent genotypes. The figure shows an extract from the AllimOptions file.

Modules 3 & 4: Estimation of the remaining mapping bias with simulated data & Estimation of allele specific expression for experimental data

Module 3 is designed to estimate the remaining mapping bias for each gene (exon) via the simulated RNA-seq reads. In the simulated data the number of reads for each transcript is identical for both parental genotypes (parental lines). Resulting expression ratios between counts for both genotypes (parental lines) should therefore be **1** for each gene (exon) in the absence of a remaining mapping bias due to the mapper. Ratios deviating from **1** indicate a remaining mapping bias and are used as a correction factor to determine statistical significance of allelic imbalance in module 5.

Module 4 is designed to estimate the total gene expression in both parental lines and allele specific gene expression in the F1 generation. The module generates an expression table for all genes and all exons in two separate files. This expression table will be subsequently used in module 5 to test for significant allelic imbalance.

Modules 3 & 4 make use of a two-genome approach to determine allele specific expression. In this approach the two “parental” genomes (one for each parental genotype / parental line) that where created for the RNA-seq read simulation in module 2 (AllimOptions_2Pexpr) or provided by the user (AllimOptions_2Pgenomes) are used as a “combined reference” (genome of parent 1 plus genome of parent 2) to map RNA-seq reads. Only the reads that

map unambiguously (no multiple equally good mapping positions) are used to determine allele specific expression. This approach excludes reads that do not span fixed SNPs as these have at least two equally good mapping locations in the "combined reference".

The input files and other parameters for modules 3& 4 can be specified in the AllimOptions file as shown in figure 5.

```
##########################################################################################
#
# ASE OPTIONS
# Step3: Measuring Allele-specific expression (ASE) with simulated as well as experimental data
#
##########################################################################################

## Provide the paths (relative to <Allim_1.0>) of the paired-end sequencing files-
#  of the two parents (parent1, parent2) and the hybrid (F1) for all present replicates.
#
## Paired-end sequencing files and insert size (fragment size - 2(read length)) should-
## be provided in following format:
# read1.fq,read2.fq,78
# The read1.fq file contains all "read 1" of the paired end reads. read2.fq contains-
# all corresponding "read 2". 78 is the insert. Make sure that the order of the paired-
# end reads is identical in both fastq files.
# The insert size is an optional parameter. Fastq files for both paired-end reads are mandatory.
#
# For each replicate provide 3 RNA-seq files 1) parent1; 2) parent2; and 3) hybrid (F1)
# In case no RNA-seq data for the parents is available,
# PARENT1_FASTQ_FILE_EXPR and PARENT2_FASTQ_FILE_EXPR should be empty.

#### Replicate 1: parent1,parent2 and hybrid fastq file data
PARENT1_FASTQ_FILE_EXPR = /Allim_1.0/test_data/parent1_read1.fastq,/Allim_1.0/test_data/parent1_read2.fastq,68
PARENT2_FASTQ_FILE_EXPR = /Allim_1.0/test_data/parent2_read1.fastq,/Allim_1.0/test_data/parent2_read2.fastq,78
HYBRID_FASTQ_FILE_EXPR = /Allim_1.0/test_data/hybrid_read1.fastq,/Allim_1.0/test_data/hybrid_read2.fastq,128
#
#### Replicate 2: parent1,parent2 and hybrid fastq file data
#PARENT1_FASTQ_FILE_EXPR =
#PARENT2_FASTQ_FILE_EXPR =
#HYBRID_FASTQ_FILE_EXPR =

#
#### Replicate 3: parent1,parent2 and hybrid fastq file data
#PARENT1_FASTQ_FILE_EXPR =
#PARENT2_FASTQ_FILE_EXPR =
#HYBRID_FASTQ_FILE_EXPR =
```

Figure 5: Input options for estimating total allele specific expression. The figure shows an extract from the AllimOptions_2Pexpr file. In AlimOptions_2Pgenomes only fastq files for the F1 hybrid have to be provided.

Module 5: Statistical test of significant allelic imbalance

G-tests of allelic imbalance for each gene/exon are provided for samples without biological replication. If biological replicates are available, Allim provides an alternative approach to determine allelic imbalance across replicates. In an ANOVA framework it is tested whether differences are present in the expression strength between both alleles in the F1 generation.

Module 5 returns p-values for each gene (exon) including multiple testing correction via the false discovery rate (FDR).

For both approaches (G-test, ANOVA) three different correction factors are implemented in Allim, namely 1) Library size normalization, 2) Mapping bias correction and 3) Rescaling of the corrected read count. These different normalization steps are explained in detail below together with examples (tables 1-4).

1) Library size normalization

Libraries are normalized by the TMM factor (trimmed mean of M-values normalization method; *Robinson and Oshlack 2010*). The factor is calculated using the bioconductor package *edgeR* (*Robinson et al. 2010*) for each parental library and the F1 library for each replicate. Individual gene counts are then corrected by the TMM factor of the respective library. Note that for each hybrid library only one TMM factor has to be calculated. This factor is used for the expression profile of both parental alleles. Table 1 provides read counts before and table 2 after the first normalization step.

Table 1: Expression table with raw read counts of two parental lines (ps88 and ps94) and the F1 hybrid for ps88 and ps94 alleles. Only the expression values of 10 out of 4604 genes are provided.

Gene_ID	ps88	ps94	ps88_hybrid	ps94_hybrid
FBgn0011563	633	4	315	3
FBgn0012690	107	173	59	59
FBgn0012695	41	18	8	18
FBgn0012699	175	205	60	62
FBgn0012706	56	74	45	33
FBgn0012708	99	135	35	54
FBgn0012709	40	52	17	28
FBgn0012711	97	88	46	32
FBgn0012714	1159	1080	557	658
FBgn0012717	2607	2520	1008	1296

Table 2: Expression table with read counts after the TMM normalization of the two parental lines (ps88 and ps94) and the F1 hybrid for ps88 and ps94 alleles. The TMM factors for the three different libraries are: TMM (ps88) = 0.9377694, TMM (ps94) = 1.084862, TMM (hybrid) = 0.9829452. Only the expression values of 10 out of 4604 genes are shown.

Gene_ID	ps88	ps94	ps88_hybrid	ps94_hybrid
FBgn0011563	593.60801	4.33945	309.627725	2.948835
FBgn0012690	100.34132	187.6812	57.993764	57.993764
FBgn0012695	38.44854	19.52752	7.863561	17.693013
FBgn0012699	164.10964	222.3968	58.976709	60.9426
FBgn0012706	52.51508	80.27982	44.232532	32.43719
FBgn0012708	92.83917	146.45643	34.403081	53.079039
FBgn0012709	37.51077	56.41285	16.710068	27.522464
FBgn0012711	90.96363	95.46789	45.215477	31.454245
FBgn0012714	1086.8747	1171.65143	547.500453	646.777914
FBgn0012717	2444.76474	2733.85333	990.808719	1273.896925

2) Mapping bias correction

The residual mapping bias after accounting for fixed SNPs between the two parental lines is accounted for via the mapping bias coefficient. This coefficient is defined as the expression ratio between both parental alleles obtained from the simulated data (module 2).

Mapping bias coefficient = readcount_simuldata(parent1) / readcount_simuldata(parent2)

The mapping bias coefficient is calculated for each gene. All read counts normalized by library size (table 2) are then multiplied by the mapping bias coefficient of the respective gene (table 3).

Table 3: Expression table with read counts after the TMM normalization & mapping bias correction of the two parental lines (ps88 and ps94) and the F1 hybrid for ps88 and ps94 alleles. The mapping bias coefficient for each gene is shown in the 6th column. Only the expression values of 10 out of 4604 genes are shown.

Gene_ID	ps88	ps94	ps88_hybrid	ps94_hybrid	Mapping bias coefficient
FBgn0011563	593.6080091	4.339449731	309.6277248	2.948835474	1.0000000
FBgn0012690	100.3413222	187.6812009	57.99376432	57.99376432	1.0000000
FBgn0012695	38.44854403	19.52752379	7.863561264	17.69301284	1.0000000
FBgn0012699	164.4542263	222.8637736	59.10054509	61.07056326	1.0020997
FBgn0012706	68.55262532	104.7964118	57.74067066	42.34315848	1.3053892
FBgn0012708	92.8391673	146.4564284	34.40308053	53.07903853	1.0000000
FBgn0012709	37.51077467	56.4128465	16.71006769	27.52246442	1.0000000
FBgn0012711	90.96362856	95.46789408	45.21547727	31.45424506	1.0000000
FBgn0012714	1086.874696	1171.651427	547.500453	646.777914	1.0000000
FBgn0012717	2445.858349	2735.076257	991.251935	1274.466774	1.0004473

3) Rescaling of the corrected read count

After applying the above two normalization factors, read counts for the different genes across libraries can be inflated or decreased depending on the values of the respective factors used. As inflation results in an unjustified increase of power in the statistical test and a lowered count in an unjustified decrease, the values have to be rescaled on a gene-wise level. This is done via the following rescaling factor.

Rescaling factor = expression value before normalization (sum over all samples) /after normalization (sum over all samples)

All corrected read counts by the two previous correction factors (table 3) are multiplied by the rescaling factor of the respective gene (table 4).

Table 4: Expression table with read counts after all three normalization steps of the two parental lines (ps88 and ps94) and the F1 hybrid for ps88 and ps94 alleles. The rescaling factor for each gene is shown in the 6th column. Only the expression values of 10 out of 4604 genes are shown.

Gene_ID	ps88	ps94	ps88_hybrid	ps94_hybrid	Rescaling factor
FBgn0011563	622.603728	4.551416993	324.7519791	3.092875991	1.048846576
FBgn0012690	98.84864517	184.8892561	57.13104934	57.13104934	0.985124004
FBgn0012695	39.12394206	19.87054981	8.001694811	18.00381332	1.017566284
FBgn0012699	162.675455	220.4532324	58.46130124	60.41001128	0.989183791
FBgn0012706	52.14788647	79.71848427	43.92324765	32.21038161	0.760698605
FBgn0012708	91.76589982	144.7633184	34.00536361	52.46541815	0.988439497
FBgn0012709	37.19686751	55.94075824	16.57023027	27.29214397	0.991631547
FBgn0012711	90.92862451	95.43115672	45.19807773	31.44214103	0.999615186
FBgn0012714	1087.251019	1172.057104	547.6900213	647.0018564	1.000346243
FBgn0012717	2440.717007	2729.326962	989.1682636	1271.787768	0.99789794

7. Allim Output / Results Files

After running Allim, the results of the Allim pipeline can be found in the output directory specified in the Allim Options file. Results are provided in the following format.

```
<OUTPUT_DIR>
        |
        - <01_fixed_SNP*1>
                |
                - <1_fixed_snp.txt>
                - <2_fixed_snp.txt >
                - <3_fixed_snp.txt >
                - .
                - .
                - .
                - <n_fixed_snp.txt*>

        - <02_simulation_data_ase>
                |
                        o   <gene-expression-table>
                        o   <exon-expression-table >

        - <03_experimental_data_ase>
                |
                - <raw_gene-expression-table>
                - <raw_exon-expression-table>

        - <04_statistical_test>
                |
```

- <corrected_ gene-expression-table with p-value*2>
- <corrected_ exon-expression-table with p-value*2>

Note:

'*1' this directory is only provided if the input option "AllimOptions_2Pexpr" was chosen.
'*2' indicates the final output files in above structure; the remaining files are intermediate output files constructed by the pipeline.

<01_fixed_SNP>:
This folder contains the identified fixed SNPs between the parental genotypes (parental lines). The number at the beginning of the file indicates in which cycle of remapping the fixed SNPs were called. "n" is a parameter that has to be provided by the user.

<02_simulation_data_ase>:
This folder contains the expression tables with the raw read counts of the simulated data for both parental genotypes (lines). Profiles are obtained for genes as well as for exons as units for which gene expression is measured.

<03_experimental_data_ase>:
This folder contains the expression tables with the raw read counts of the experimental data for all replicates of both parental genotypes (lines) and the F1 generation. Profiles are obtained for genes as well as for exons as units of expression.

<04_statistical test>:
This folder contains the read counts of the experimental data, which were normalized and corrected for the residual mapping bias. Profiles for all replicates of both parental genotypes (lines) and the F1 generation are obtained for genes as well as for exons as units of expression.
The remaining two files provide the results of the significance testing for allelic imbalance for the F1 generation.

8. Benchmark of the Allim Pipeline

We have run the Allim pipeline with the following data set of RNA-seq reads from *Drosophila pseudoobscura* to benchmark the runtime of Allim. The reference genome release 2.23 was taken from Flybase and an improved annotation file of *D. pseudoobscura* was taken

from (*Palmieri et al 2012*). The GTF file with the gene annotation contained 17,112 gene models.

RNA-seq reads (Illumina, GA II):

Parent1 reads: 2 million 100 bp paired-end reads with 68 bp average insert size

Parent2 reads: 2 million 100 bp paired-end reads with 78 bp average insert size

F1 reads: 2 million 100 bp paired-end reads with 98 bp average insert size

The Allim run simulated the following amount of sequence reads:

Parent1 reads: 10.26 million 100 bp paired-end reads with 78 bp insert size

Parent2 reads: 10.26 million 100 bp paired-end reads with 78 bp insert size

Table 5: Benchmarks for processing time for each module of Allim

S.No.	Module	Time (min:sec)
1*	Identification of fixed SNPs	95:00
2	Computer simulation of RNA-seq reads with fixed SNPs	20:00
3	Estimation of allele specific expression and residual mapping bias with simulated data	34:00
4	Estimation of allele specific expression with experimental data	24:00
5	Statistical test of allelic imbalance	3:00

* This step will be skipped when RNA-seq data for parent1 and parent2 not available.

All benchmarks have been done on a Mac OS X 10.6.8, 2x2.8 GHz Quad-Core Intel Xeon, with 4 GB of RAM using 4 processors (CPU).

9. Validation

We validated the performance of our pipeline with experimental as well as simulated RNA-seq reads. The experimental data consisted of paired-end RNA-seq reads from males and females of two different isofemale lines of *Drosophila pseudoobscura* (Table 6; *Palmieri et al 2012*).

Table 6: Number of paired-end RNA-seq reads of *D. pseudoobscura*, which were used for validation. (ps88 and ps94 stand for two different isofemale lines.)

**Samples**	**Read pairs (in million)**	**Insert Size (bp)**	**Read length (bp)**
ps88 males	79.21	78	100
ps88 females	80.00	78	100
ps94 males	79.21	128	100
ps94 females	80.00	68	100

The four libraries were given as input to Allim to call fixed differences between both isofemale lines (different sexes were used as biological replicates). Module 1 of the pipeline identified154,920 fixed SNPs present in a total of 9,626 genes. The following Allim parameters were used to call the fixed SNPs:
CALCULATE_FIXED_SNP_ITERATION = 2
MINIMUM_COVERAGE = 2
FIXED_ALLELE_FREQURNCY = 1.0
MINIMUM_BASE_QUALITY = 20
MINIMUM_MAPPING_QUALITY = 20
The reference genome release 2.23 was taken from Flybase and an improved annotation file of *D. pseudoobscura* was taken from (*Palmieri et al 2012*). The GTF file with the gene annotation contained 17,112 gene models.

Assignment of reads to the correct parental line:

To test the accuracy of Allim to identify the parental origin of a read, ASE expression profiles were determined for experimental and simulated data:

1. Experimental data:
a) pooled RNA-seq reads from libraries ps88 males and ps94 males (Table 6)
b) pooled RNA-seq reads from libraries ps88 females and ps94 females (Table 6)

In contrast to RNA-seq data of individuals from the F1 generation, the parental origin of the pooled reads from both lines is known. Therefore, the percentage of reads that were assigned to the correct parental line by Allim could be determined. The results show that between 98.60% and 99.97% of the reads were assigned to the correct parental line by Allim (Table 7).

The marginal difference between the success rates of both lines is due to the fact that ps94 is a derivative of the strain for which the reference genome sequence was provided to Allim. For this reason reads originating from ps94 that span polymorphic sites, which did not meet the threshold for calling fixed SNPs, are more easily mapped than reads originating from ps88. Since we used the most extreme case for a reference bias, the small bias observed suggests that under less extreme settings, Allim will provide almost no bias caused by the reference genome.

2. Simulated data:

RNA-seq reads were simulated with the procedure described in module 2 based on the 154,920 fixed SNPs determined for the experimental data previously. (Approach: For both parental genomes the same number of RNA-seq reads were simulated for the identical genomic positions.) For the simulation the following Allim parameters were used:

ASCII_QUALITY_LETTER = e

READ_LENGTH = 100

INSERT_SIZE = 78

The results show that 99.99% of the simulated reads were assigned to the correct parental line by Allim (Table 7). As the reads were directly simulated based on the reference genome with only fixed differences edited, this remaining error occurs during the mapping with GSNAP (possibly for reads spanning splice junctions).

Table 7: RNA-seq data sets used to test accuracy of Allim to identify the parental origin of a read.

**Dataset**	**# Correctly identified reads, ps88 (%)**	**# Correctly identified reads, ps94 (%)**
Pooled reads from females of both lines	99.97	98.96
Pooled reads from males of both lines	99.96	98.60
Simulated reads for both parental genomes	99.99	99.99

Improvement of mapping quality via modification of the reference genome

We validated the improvement of the mapping quality via reference modification by mapping

RNA-seq reads (experimental & simulated data) to the original as well as the modified genomes. Mapping success was then compared between both approaches. The mapping success varies between 85.97% and 92.15% between the different data sets. However, in all data sets an increase in mapping success after editing of the genomes between 0.07% and 0.32% can be detected (Table 8).

Table 8: Improvement of mapping success via genome modification.

RNA-Seq data	Total number of single reads		#Mapped single reads [%]				Improvement [% of total number of reads]	
			Before genome modification		After genome modification			
	# reads p88	#reads p94	ps88	ps94	ps88	ps94	ps88	ps94
Female data	79,981,000	79,998,000	91.18	92.15	91.49	92.22	0.31	0.07
Male data	76,877,000	79,207,000	85.97	86.73	86.19	87.05	0.22	0.32
Simulation data	122,682,000	122,682,000	90.94	90.96	91.05	91.03	0.11	0.07

We validated the performance of our pipeline with experimental as well as simulated RNA-seq reads. The experimental data consisted of paired-end RNA-seq reads from males and females of two different isofemale lines of *Drosophila pseudoobscura* (Table 6, *Palmieri et al 2012*).

10. Contact Information

Prof. Dr. Christian Schlötterer
christian.schloetterer@vetmeduni.ac.at

Ram Vinay Pandey
ramvinay.pandey@vetmeduni.ac.at

Susanne U. Franssen
susanne.franssen@vetmeduni.ac.at

11. References:

1. Wu TD, Nacu S (2010) Fast and SNP-tolerant detection of complex variants and splicing in short reads. Bioinformatics 26: 873-881.
2. Robinson MD, Oshlack A (2010) A scaling normalization method for differential expression analysis of RNA-seq data. Genome Biol. 11(3):R25
3. Robinson MD, McCarthy DJ, Smyth GK (2010) edgeR: a Bioconductor package for differential expression analysis of digital gene expression data.

Bioinformatics 26(1):139-140.
4. Palmieri N, Nolte V, Suvorov A, Kosiol C, Schlötterer C (2012) Evaluation of Different Reference Based Annotation Strategies Using RNA-Seq – A Case Study in *Drososphila pseudoobscura*. *PLoS One*, **7**, e46415.
